# Hepatitis E virus seroprevalence in pets in the Netherlands and the permissiveness of canine liver cells to the infection

**DOI:** 10.1186/s13620-020-00158-y

**Published:** 2020-04-02

**Authors:** Yunlong Li, Changbo Qu, Bart Spee, Ruyi Zhang, Louis C. Penning, Robert A. de Man, Maikel P. Peppelenbosch, Hille Fieten, Qiuwei Pan

**Affiliations:** 1 Department of Gastroenterology and Hepatology, Erasmus MC-University Medical Center, room Na-1005, ‘s-Gravendijkwal 230, NL-3015 CE Rotterdam, The Netherlands; 2 Department of Clinical Sciences of Companion Animals, Faculty of Veterinary Medicine, Utrecht University, Utrecht, The Netherlands

**Keywords:** HEV, Dog, Cat, Horse, Seroprevalence, Infection

## Abstract

Hepatitis E virus (HEV) as an emerging zoonotic pathogen causes a major public health issue. Transmission from domestic, wildlife and zoo animals to human has been widely reported. Whether pets also serve as reservoirs remains an intriguing question. In this study, we found the sero-positive rates of HEV-specific antibodies in pet dogs, cats and horses of 18.52% (30/162), 14.89% (7/47) and 18.18% (4/22) in the Netherlands. Although HEV viral RNA was not detected in these animals, we have demonstrated that dog liver cells are susceptible to HEV infection in vitro. These results call more attention to address the potential role of pets in the zoonotic transmission of HEV.

## Introduction

Hepatitis E virus (HEV) as a single-stranded positive-sense RNA virus belongs to the *Hepeviridae* family and is the causative agent for hepatitis [1]. Although there is only one serotype, HEV strains are currently classified into eight genotypes, of which four are the most important for human diseases. Genotype 1 and 2 are known to infect human only, and mainly cause acute infection. On the contrary, genotype 3 and 4 are well-recognized zoonotic strains circulating among a broad spectrum of animal species. Both domesticated and wild animals have been recognized as potential reservoirs [2–4]. Indeed, evidence has indicated that companion animals including dogs and cats might be accidental hosts for HEV and might constitute a source for HEV transmission to human [5, 6]. A very recent study in South Korea has reported the seroprevalence of HEV in pet dogs and pet veterinarians of 28.2 and 5.0%, respectively [7]. Genotype 3 strains have also been identified in horses, and appeared closely related to human isolates from the same region [8].

In Europe, the increase in autochthonous cases of hepatitis E mainly attributes to the zoonotic transmission of genotype 3 HEV. Although consumption of raw or undercooked pork meat and liver has been recognized as the common source of infection, other unknown transmission routes may also be important in contributing to this growing public health burden in Europe [9]. Therefore, we aimed to investigated HEV seroprevalence among pet dogs, cats and horses in the Netherlands. Furthermore, we have evaluated the permissiveness of canine liver cells to human genotype 3 HEV in experimental settings.

## Materials and methods

Serum samples from pet dog (*n* = 162), cat (*n* = 47) and horse (*n* = 22) were collected from May to September in 2017, and fecal samples from pet dog (*n* = 19) were collected in September of 2017, at the Department of Clinical Sciences of Companion Animals, Faculty of Veterinary Medicine, Utrecht University, The Netherlands. Samples were stored at − 80 °C until analysis.

Total HEV-specific antibodies in sera were detected by the species-independent double-antigen sandwich ELISA (WANTAI HEV-Ab #WE-7396) according to the manufacturer’s instructions. A SYBR green-based quantitative real-time PCR (qRT-PCR) array was used to quantify HEV RNA in the serum and fecal samples (primers are listed in Supplementary table 1). All anti-HEV antibody positive serum samples were tested individually, whereas every ten negative samples were pooled with 30 μL of each for HEV RNA detection. Fecal samples were tested individually. RNA was isolated with a Macherey-Nucleo Spin RNA kit. cDNA was synthesized from total RNA using a cDNA Synthesis Kit (TAKARA BIO INC). The cDNA was amplified for 50 cycles and quantified according to the manufacturer’s instructions.

The human hepatoma cell line (Huh7.5) and dog liver cancer cell line (BDE) [10] were cultured in DMEM supplemented with 10% volume/volume (v/v) fetal calf serum, 100 IU/mL penicillin and 100 μg/mL streptomycin. A plasmid construct containing the full-length HEV genome (genotype 3 Kernow-C1 p6 clone, GenBank accession number JQ679013) or a construct containing subgenomic HEV sequence in which ORF2 (Open Reading Frame 2) was replaced by a Gaussia luciferase reporter gene (p6-Luc) was used to generate HEV genomic RNA by using the Ambion mMESSAGE nMACHINE in vitro RNA transcription kit. Cells were electroporated with p6 full-length HEV RNA or p6-Luc subgenomic RNA to generate infection and luciferase-based replicon models, respectively. For the p6 infectious model, qRT-PCR was used to quantify genomic RNA. An HEV plasmid-based standard curve was constructed as a reference to assess and quantify the copy number of HEV genome (Supplementary Figure 1). HEV ORF2 protein was detected by immunofluorescent staining. For the p6-Luc replicon model, the activity of secreted luciferase in the cell culture medium was measured by the BioLux Gaussia Luciferase Flex Assay Kit (New England Biolabs, MA) and quantified with a LumiStar Optima luminescence counter (BMG LabTech, Offenburg, Germany).

## Results and discussion

To investigate the prevalence of HEV in domestic pets in the Netherlands, we have collected serum samples from 162 dogs, we found that 18.52% (30/162) dogs were positive for anti-HEV antibodies in serum. Literature data on the seroprevalence of HEV antibodies in dogs ranges from 0.8% in UK [11], 28.2% in South Korea [7], 13.54 to 36.55% in different regions of China [6, 12–14], and 56.6% in Germany [15]. Genomic HEV RNA was not detectable in these serum samples by qRT-PCR. From the 162 dogs, there were 19 fecal samples available. Although 4 of 19 matched sera were positive for anti-HEV antibodies, HEV RNA was not detected in any of the fecal samples (Table 1). Dogs experimentally infected with swine HEV have been shown to induce an antibody response but no HEV RNA was detected in serum post-inoculation [16]. HEV RNA was not detectable in any of those previous studies as well as in our study.

**Table 1:** Detection of anti-HEV total antibodies by ELISA and HEV RNA by PCR in serum and fecal samples collected from pet dogs, cats and horses

Animal Species (Serum)	ELISA (Total antibodies)	HEV RNA
	Positive/total (%)	Positive/total (%)
Dog	30/162 (18.52)	0/162 (0)
Cat	7/47 (14.89)	0/47 (0)
Horse	4/22 (18.18)	0/22 (0)

Remark: HEV RNA in dog fecal samples; positive /total (%): 0/19 (0)

**Fig. 1:**
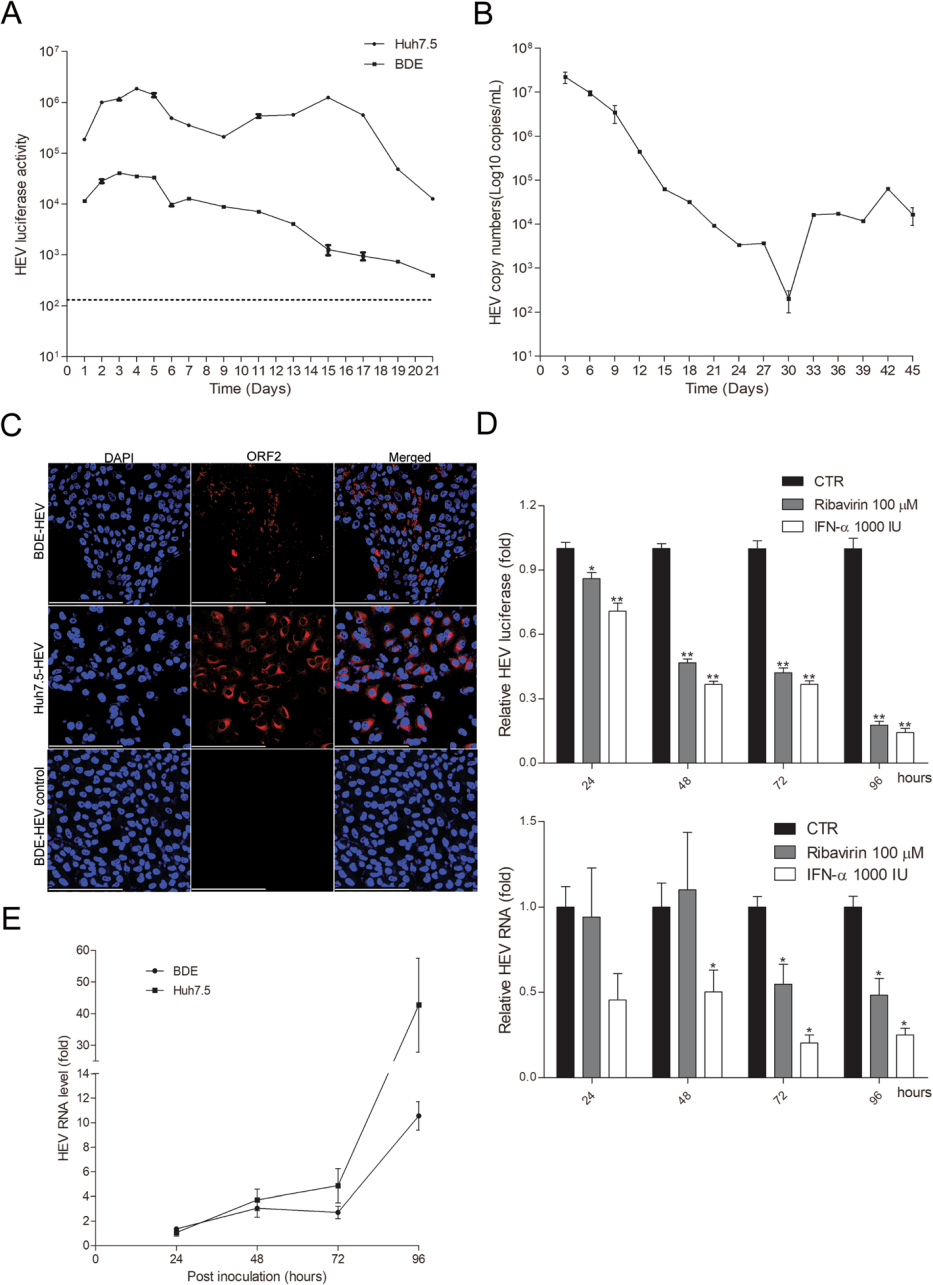
Canine liver cells support the full life-cycle of HEV infection. **a**, In BDE cells upon electroporation of the subgenomic HEV RNA with luciferase reporter, HEV luciferase activity (representing virus replication level) was sustained for 21 days (mean ± SEM; *n* = 2). **b**, Upon electroporation of the full-length genomic HEV RNA, BDE cells support long-term production of HEV in the medium for 45 days as quantification of the viral genome by qRT-PCR and calculated as copy numbers (mean ± SEM; *n* = 2). **c**. Immunofluorescence staining of viral protein ORF2 (red) in BDE cells. BDE-HEV cells incubated with the matched IgG control antibody serves as negative control, and HEV infected Huh7.5 cells serves as positive control. DAPI (blue) was applied to visualize nuclei. (40× oil immersion objective; Scale bar, 200 μm). **d**, Anti-HEV activity of ribavirin and interferon-α (IFN-α) in BDE cell based HEV models. Treatment of ribavirin or IFN-α for 24, 48, 72 or 96 h in the subgenomic model determined by luciferase activity (mean ± SEM, *n* = 5. CTR, non-treatment control.), and in the infectious model determined by viral RNA (mean ± SEM, *n* = 4. CTR, non-treatment control). *P < .05; **P < .001; ***P < .0001. **e**, BDE and Huh7.5 cells are permissive for secondary infection with inoculation of BDE cells produced HEV viral particles as determined by qRT-PCR at 24, 48, 72 and 96 h post-inoculation (mean ± SEM; n = 2). The level of HEV infection in Huh7.5 cells for 24 h serves as 1. GAPDH serves as a reference gene

We next investigated whether canine liver cells are permissive for HEV infection in cell culture models. Electroporation of subgenomic HEV RNA (p6-Luc; human genotype 3 strain) into the BDE dog liver cell line resulted in production of secreted luciferase as monitored up to 21 days. Although the luciferase activity was gradually decreased overtime and the overall activity is lower compared to that in the human hepatoma Huh7.5 cells, these results indicate that dog liver cells can support HEV replication (Fig. 1a). Of note, Huh7.5 cells are highly permissive and widely used for modeling HEV infection in vitro [17]. Consistently, electroporation of the full-length genomic HEV RNA into BDE cells resulted in long-term production of HEV in the supernatant as monitored over 45 consecutive days (Fig. 1b). Furthermore, HEV ORF2 protein was expressed in these cells detected by immunofluorescent staining (Fig. 1c).

We confirmed the anti-HEV activity of interferon-α and ribavirin in Huh7.5 based HEV subgenomic and infectious models (Supplementary Figure 2). Consistently, we found that interferon-α effectively inhibited viral replication in BDE cells in both HEV models. The inhibitory effects of ribavirin were potent in the subgenomic model, but only effective at later time points (72 and 96 h) in the infectious model (Fig. 1d). To verify whether produced HEV are infectious, we collected supernatant from full-length HEV RNA electroporated BDE cells with a HEV genomic titer of 7.4 × 10^6^ copies/mL and inoculated the naïve BDE and Huh7.5 cells. We have demonstrated the replication of HEV in both cell lines by quantification of intracellular viral RNA (Fig. 1e) and immunofluorescent staining of ORF2 protein (Supplementary Figure 3). This also support that dog liver cells are permissive for the entry of infectious HEV particles. Collectively, we have demonstrated that dog liver cells are permissive for human genotype 3 HEV, and support the full life-cycle of the infection. Nevertheless, this human HEV strain is a cell culture adapted strain that may not fully recapitulate the clinical strains.

In addition, we found that 14.89% (7/47) of cats and 18.18% (4/22) of horses were positive for anti-HEV antibodies. The anti-HEV seroprevalence rates in cats have been reported to be 6.28% in China [12], 8.1% in Korea [16], and 33% in Japan [5], but our data are the first to come from Europe. Up to date, only two studies have investigated the prevalence of HEV infection in horses. 13% (26/200) of anti-HEV antibody positivity and 2% (4/200) HEV RNA positivity were found in work horses in Egypt [8]. In a study from China, 16.3% (8/49) of horses were found to be positive of anti-HEV antibody and HEV RNA was detected in one horse [14]. Overall, our data suggest that companion animals are frequently exposed to HEV and thus potentially infectious for humans in close contact with these animals. In apparent agreement, when comparing with the general population, veterinarians and dog farm staff who are frequently exposed to dogs have significantly higher rates of anti-HEV antibody positivity [6].

In summary, this study has pioneered the survey of HEV infection in pets in the Netherlands. We found substantial positive rates of anti-HEV antibodies in dogs, cats and horses, but viral RNA was not detected. However, the sample size was relatively small and may not be fully representative for the country. In addition, dietary factors may differ between countries with different input of raw animal meat in dog and cat food. Importantly, our experimentation in cell culture has demonstrated that dog liver cells are permissive for human genotype 3 HEV infection. Thus, further investigation into the prevalence and the potential of zoonotic transmission from pets is urged.

## Supplementary information

**Additional file 1: Table S1.** qRT-PCR primer sequences. **Figure S1.** qRT-PCR determined standard curve. HEV plasmid based standard curve is generated by plotting the log copy number versus the cycle threshold (CT) value. **Figure S2.** Potent anti-HEV activity of ribavirin and interferon-α (IFN-α) in Huh7.5 cell model. Treatment of ribavirin or IFN-α for 24, 48, 72 or 96 h in the subgenomic model determined by luciferase activity (mean ± SEM, *n* = 5. CTR, non-treatment control.), and in the infectious model determined by viral RNA (mean ± SEM, *n* = 4. CTR, non-treatment control). *P < .05; **P < .001; ***P < .0001. **Figure S3.** Immunofluorescence staining of viral protein ORF2 (red) in BDE cells, upon infection of 24 h, 48 h, 72 h and 96 h. BDE-HEV cells incubated with the matched IgG control antibody serves as negative control, and HEV infected Huh7.5 cells serves as positive control. DAPI (blue) was applied to visualize nuclei. (40× oil immersion objective; Scale bar, 200 μm).

## Data Availability

The data that support the findings of this study are available from the corresponding author upon reasonable request.

## Abbreviations

HEV: Hepatitis E virus
qRT-PCR: quantitative real-time PCR
ORF2: Open Reading Frame 2

## References

[1] Smith DB, Simmonds P, Jameel S, Emerson SU, Harrison TJ, G. International Committee on Taxonomy of Viruses Hepeviridae Study (2014). Consensus proposals for classification of the family Hepeviridae. J Gen Virol.

[2] Meng XJ (2016). Expanding host range and cross-species infection of hepatitis E virus. PLoS Pathog.

[3] Meng XJ (2013). Zoonotic and foodborne transmission of hepatitis E virus. Semin Liver Dis.

[4] Temmam S, Besnard L, Andriamandimby SF, Foray C, Rasamoelina-Andriamanivo H, Heraud JM (2013). High prevalence of hepatitis E in humans and pigs and evidence of genotype-3 virus in swine, Madagascar. Am J Trop Med Hyg.

[5] Okamoto H, Takahashi M, Nishizawa T, Usui R, Kobayashi E (2004). Presence of antibodies to hepatitis E virus in Japanese pet cats. Infection.

[6] Zeng MY, Gao H, Yan XX, Qu WJ, Sun YK, Fu GW (2017). High hepatitis E virus antibody positive rates in dogs and humans exposed to dogs in the south-west of China. Zoonoses Public Health.

[7] Lyoo KS, Yang SJ, Na W, Song D (2019). Detection of antibodies against hepatitis E virus in pet veterinarians and pet dogs in South Korea. Ir Vet J.

[8] Saad MD, Hussein HA, Bashandy MM, Kamel HH, Earhart KC, Fryauff DJ (2007). Hepatitis E virus infection in work horses in Egypt. Infect Genet Evol.

[9] The L (2017). Growing concerns of hepatitis E in Europe. Lancet.

[10] Boomkens SY, Spee B, Ijzer J, Kisjes R, Egberink HF, van den Ingh TS (2004). The establishment and characterization of the first canine hepatocellular carcinoma cell line, which resembles human oncogenic expression patterns. Comp Hepatol.

[11] McElroy A, Hiraide R, Bexfield N, Jalal H, Brownlie J, Goodfellow I (2015). Detection of hepatitis E virus antibodies in dogs in the United Kingdom. PLoS One.

[12] Liang H, Chen J, Xie J, Sun L, Ji F, He S (2014). Hepatitis E virus serosurvey among pet dogs and cats in several developed cities in China. PLoS One.

[13] Liu J, Zhang W, Shen Q, Yang S, Huang F, Li P (2009). Prevalence of antibody to hepatitis E virus among pet dogs in the Jiang-Zhe area of China. Scand J Infect Dis.

[14] Zhang W, Shen Q, Mou J, Gong G, Yang Z, Cui L (2008). Hepatitis E virus infection among domestic animals in eastern China. Zoonoses Public Health.

[15] Dahnert L, Conraths FJ, Reimer N, Groschup MH, Eiden M (2018). Molecular and serological surveillance of hepatitis E virus in wild and domestic carnivores in Brandenburg, Germany. Transbound Emerg Dis.

[16] Song YJ, Jeong HJ, Kim YJ, Lee SW, Lee JB, Park SY (2010). Analysis of complete genome sequences of swine hepatitis E virus and possible risk factors for transmission of HEV to humans in Korea. J Med Virol.

[17] Xu L, Wang W, Li Y, Zhou X, Yin Y, Wang Y (2017). RIG-I is a key antiviral interferon-stimulated gene against hepatitis E virus regardless of interferon production. Hepatology.

