# Supplementary material for: Hepatitis E virus seroprevalence in pets in the Netherlands and the permissiveness of canine liver cells to the infection
Source: Ir Vet J. 2020 Apr 2;73:6. doi: 10.1186/s13620-020-00158-y (PMC7119158; doi:10.1186/s13620-020-00158-y)
Supplement: Supplementary file 1 — Additional file 1: Table S1. qRT-PCR primer sequences. Figure S1. qRT-PCR determined standard curve. HEV plasmid based standard curve is generated by plotting the log copy number versus the cycle threshold (CT) value. Figure S2. Potent anti-HEV activity of ribavirin and interferon-α (IFN-α) in Huh7.5 cell model. Treatment of ribavirin or IFN-α for 24, 48, 72 or 96 h in the subgenomic model determined by luciferase activity (mean ± SEM, n = 5. CTR, non-treatment control.), and in the infectious model determined by viral RNA (mean ± SEM, n = 4. CTR, non-treatment control). *P < .05; **P < .001; ***P < .0001. Figure S3. Immunofluorescence staining of viral protein ORF2 (red) in BDE cells, upon infection of 24 h, 48 h, 72 h and 96 h. BDE-HEV cells incubated with the matched IgG control antibody serves as negative control, and HEV infected Huh7.5 cells serves as positive control. DAPI (blue) was applied to visualize nuclei. (40× oil immersion objective; Scale bar, 200 μm). [file 13620_2020_158_MOESM1_ESM.pdf]

Supplementary information to

Hepatitis E virus seroprevalence in pets in the Netherlands and the permissiveness of canine liver cells to the infection

Yunlong Li, Changbo Qu, Bart Spee, Ruyi Zhang, Louis C. Penning, Robert A. de Man, Maikel P. Peppelenbosch, Hille Fieten, Qiuwei Pan

Supplementary Table 1.

Table 1. qRT-PCR primer sequences

Primer ID	Sequence (5'-3')
HEV sense	GGTGGTTTCTGGGGTGAC
HEV anti sense	AGGGGTTGGTTGGATGAA
Human GAPDH sense	GTCTCCTCTGACTTCAACAGCG
Human GAPDH antisense	ACCACCCTGTTGCTGTAGCCAA
Dog GAPDH sense	GATGGGCGTGAACCATGAG
Dog GAPDH antisense	TCATGAGGCCCTCCACGAT

Supplementary Figure 1.

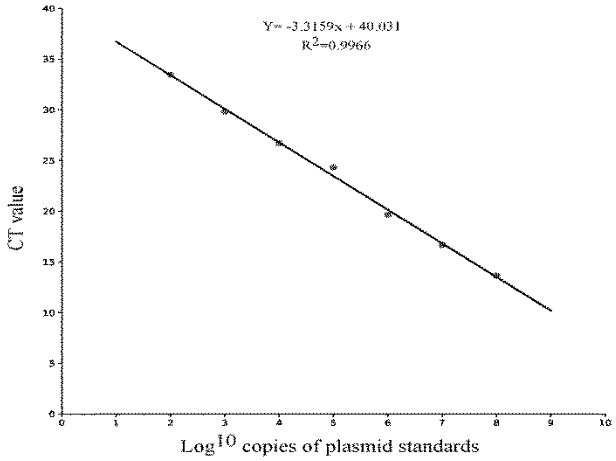

Supplementary Figure 1. qRT-PCR determined standard curve. HEV plasmid based standard curve is generated by plotting the log copy number versus the cycle threshold (CT) value.

Supplementary Figure 2.

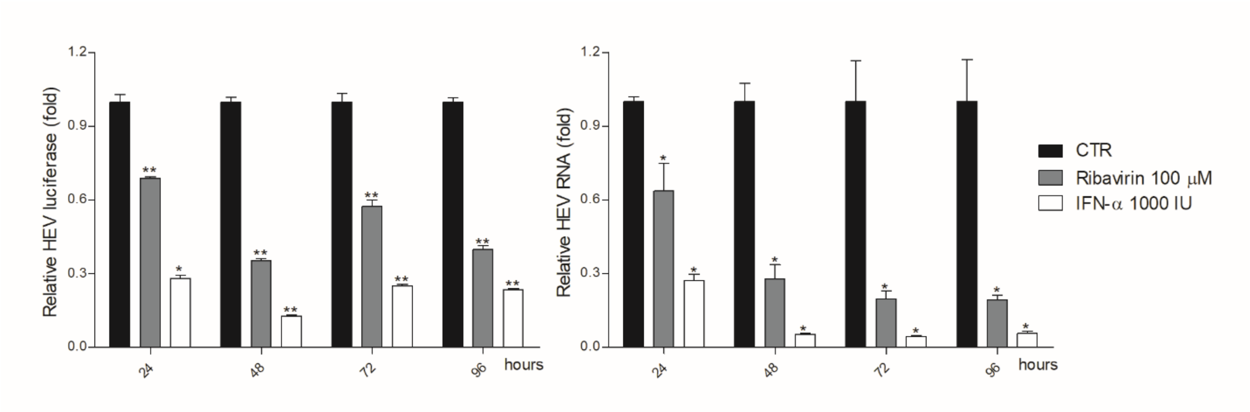

Supplementary Figure 2. Potent anti-HEV activity of ribavirin and interferon-α (IFN-α) in Huh7.5 cell model. Treatment of ribavirin or IFN-α for 24, 48, 72 or 96 hours in the subgenomic model determined by luciferase activity (mean ± SEM, n = 5. CTR, non-treatment control.), and in the infectious model determined by viral RNA (mean ± SEM, n = 4. CTR, non-treatment control). *P < .05; **P < .001; ***P < .0001.

Supplementary Figure 3.

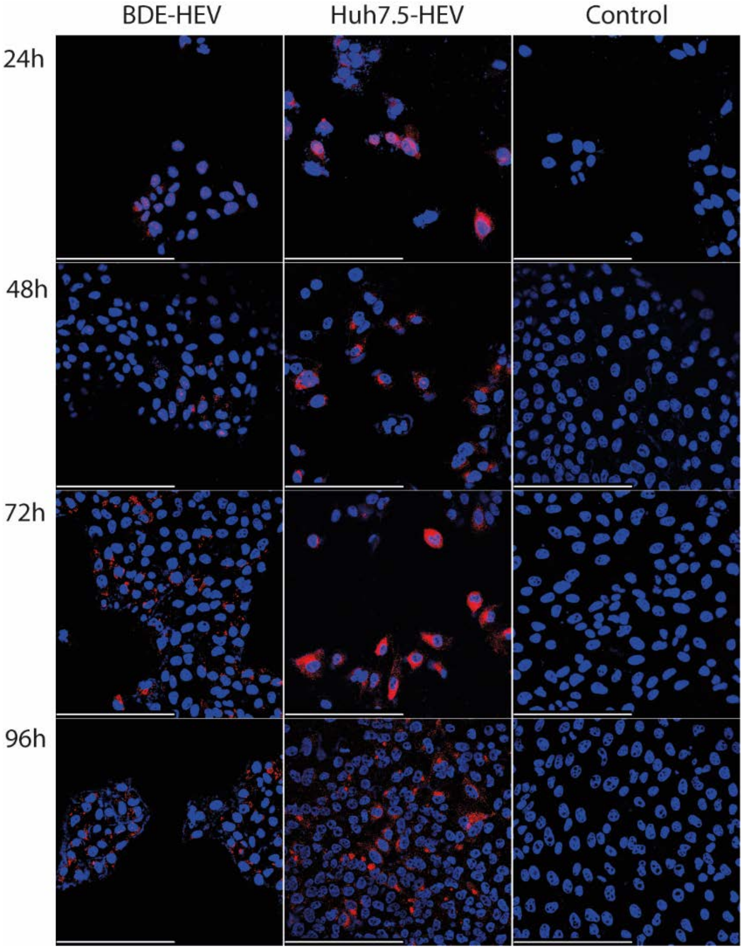

Supplementary Figure 3. Immunofluorescence staining of viral protein ORF2 (red) in BDE cells, upon infection of 24h, 48h, 72h and 96h. BDE-HEV cells incubated with the matched IgG control antibody serves as negative control, and HEV infected Huh7.5 cells serves as positive control. DAPI (blue) was applied to visualize nuclei. (40× oil immersion objective; Scale bar, 200 μm).

Supplementary Methods

Electroporation of HEV RNA. The BDE cells were collected and centrifuged for 3 minutes at 1500 rpm, 4°C. The supernatant was removed and the cells were washed with 4 mL Opti-MEM (Thermo Scientific, The Netherlands) by centrifuging for 5 minutes at 1500 rpm, 4°C, 3 times. The cell pellet was resuspended in 100 μL Opti-MEM and mixed with p6 full-length HEV RNA or p6-Luc subgenomic RNA. Electroporation was performed with Bio-Rad's electroporation systems using the protocol of a designed program (600 volts, pulse length 0.5, number 1, 4 mm cuvette).

Immunofluorescence. Cells grown on coverslips were fixed with 4% (w/v) paraformaldehyde (PFA) for 10 min at RT. After three washes with PBS buffer, cells were permeabilized with 0.1% (v/v) Triton X-100 for 10 min and washed three times with PBS. Block for 1h at Room Temperature with blocking solution (5% Normal Donkey Serum, 1% Bovine Serum Albumin, 0.2% TRITON X in 1x PBS). Cells were then incubated with primary anti-HEV ORF2 (1:200) antibody (aa 434-457, clone 1E6, Millipore, Amsterdam-Zuidoost, the Netherlands) at 4°C overnight. The control group was incubated with the matched mouse IgG 2b antibody (1:200) (InvivoGen) at 4°C overnight. Remove excess primary antibodies and wash the cells for three times with PBS, inoculate the cells with Anti-mouse-Alexa Fluor® 594-Conjugate antibody (Cell Signaling Technology) (1:5000) for 1 hour at room temperature. Nucleus was stained with 4,6-diamidino-2-phenylindole (DAPI) for 10 min at room temperature. Images were detected with confocal electroscope (lens: 40×).

Statistical analysis. Statistical analysis was performed using the nonpaired, nonparametric test with the Mann-Whitney test (GraphPad Prism version 5.01; GraphPad Software). P-values less than 0.05 were considered statistically significant.
